# Biofilm and Rivers: The Natural Association to Reduce Metals in Waters

**DOI:** 10.3390/toxics10120791

**Published:** 2022-12-15

**Authors:** Nicoletta Guerrieri, Laura Fantozzi, Andrea Lami, Simona Musazzi, Martina Austoni, Arianna Orrù, Laura Marziali, Gigliola Borgonovo, Leonardo Scaglioni

**Affiliations:** 1 National Research Council, Water Research Institute, Largo Tonolli 50, I-28922 Verbania Pallanza, Italy; 2 National Research Council, Water Research Institute, Via del Mulino 19, I-20861 Brugherio, Italy; 3 DeFENS Department of Food, Environmental and Nutritional Sciences, Via Celoria 2, I-20133 Milano, Italy

**Keywords:** biofilm, water quality, oxidative stress, extruded polymeric substance, inductively coupled plasma mass spectrometry, metals, mercury, arsenic

## Abstract

This article focuses on a very peculiar habitat, the thin biofilm that covers the surface of rocks, cobbles, sediment grains, leaf litter, and vegetation on a riverbed. Species composition changes over time and depends on environmental conditions and perturbation of water quality. It provides several ecosystem services, contributing to the biogeochemical fluxes and reducing contamination by absorbing the pollutants. Biofilm into the Toce River (Ossola Valley, Piedmont, Italy) was investigated to assess its capacity to accumulate the metals and macroions from the water column. In this preliminary work, we investigated three sample points, in two different seasons. The community composition of biofilm was determined via morphological analysis (diatoms and non-diatoms algal community). We characterize the biofilm, a community of different organisms, from different perspectives. In the biofilm, Hg was analyzed with an automated mercury analyzer, other metals and macroions with inductively coupled plasma mass spectrometry (ICP-MS) (Al, As, Ba, Ca, Cr, Cu, Fe, K, Mg, Mn, Ni, P, Pb, and Zn), and the carotenoid and chlorophyll composition of the photosynthetic organism with HPLC analysis for the primary producers. The results evidence a seasonal pattern in metals and macroions levels in the biofilm, and a significant difference in the biofilm community and in carotenoid composition, suggesting the utility of using the biofilm as an additional bioindicator to monitor the water quality of the river.

## 1. Introduction

Biofilm in rivers is an organized three-dimensional structure community of microorganisms, bacteria, archaea, microalgae, fungi, protozoa, and even metazoa. Biofilm grows on submerged solid substrates in aquatic environments, where the dynamic water flow modifies its physical structure and functionality, generating a complex structure [1]. Photosynthetic processes are carried out by primary producers, microalgae, and cyanobacteria, while biofilm-associated fungi, bacteria, and heterotrophic decomposers contribute to organic matter decomposition and circulation of essential nutrients in aquatic ecosystems [2,3]. Biofilms represent an important food resource for many invertebrates in freshwater ecosystems, assuming an important role in the transfer of contaminants into the food chain [4].

In aquatic ecosystems, biofilms adsorb, retain, amplify, and transform organic substances and nutrients in the matrix, and extruded polymeric substances (EPSs) can stabilize the structure [1]. EPSs include a wide range of different organic macromolecules, primarily polysaccharides, between 40% and 90%, but also glycoproteins and lesser amounts of lipids, nucleic acids, and proteins [5]. Biofilm has the capability to absorb contaminants through multiple mechanisms, including electrostatic interactions, cation exchange, complexation, and hydrophobic and micropore filling properties. The adsorption and degradation capacity of biofilms have been widely used for monitoring anthropogenic pollutants, such as heavy metals (e.g., copper, zinc, and cadmium), hydrophobic organic pollutants, and emerging contaminants [6]. Heavy metal contamination is a major environmental issue in river basins, and much work has been undertaken illustrating the origins, dispersal, and fate of these contaminants. In addition to the traditional measurement of contamination levels in sediments [7,8,9,10], many studies highlight the importance of biofilms as a medium for the assessment of pollutants in freshwater environments. This type of investigation allows the study of the spatial variability of contaminants and can provide more information on the risk of river ecosystems, as biofilm is intensively grazed by aquatic organisms at a higher trophic level [11,12,13].

This preliminary study aimed to integrate and improve pollution monitoring practices using biofilm as a bioindicator. As a test case, we selected an area located along the Toce River, in the Ossola Valley (Italian Central Alps), which is still heavily impacted by industrial development. A large part of the Ossola Valley develops on high hills or mountains, and its morphology is the result of the action of glaciers and erosion by rivers. The control of water flow through dams, necessary to supply electricity, has reduced periodic flooding but was unable to cancel the effects of intense rainfall, due to climate change, which most frequently affects the whole Ossola Valley. During the industrialization phase (1920–1990), the Ossola Valley area experienced its greatest economic growth, with a strong impact on the environment, with consequences still present today. In the early 1900s and, in particular, between the two World Wars, small and large industries settled in the area, especially chemical plants, attracted by the availability of low-cost electricity and watercourses in which to discharge production residues. Among them, an industrial plant operating from the First World War period was the main cause of chemical pollution in the study area [14,15]. The industrial discharges were released into the Toce River, reaching up to Lake Maggiore, one of the main Italian lakes. Therefore, the contamination had strong implications for the biota of the whole lake basin and, potentially, for human health. A large amount of data collected from 2000 until today in the framework of the monitoring programs of the International Commission for the Protection of the Italian-Swiss Waters (CIPAIS; www.cipais.org; accessed on 16 November 2022) [14,16] highlights the persistence of some pollutants in sediments and biota of the Lake Maggiore and Toce River, such us DDT and its derivatives (DDD and DDE), metals such as arsenic and mercury (Hg), PCB, and dioxin. With regard to Hg contamination, data show that, even if the activity of the chlor-alkali plant was drastically reduced in the late 1990s and finally stopped at the end of 2017, Hg contamination persists in the sediments and biota of the aquatic ecosystem, showing that active sources of Hg are still present, such as soil leaching and/or atmospheric deposition [17]. Since 2017, soil restoration has been in progress in the industrial area. 

Considering that for efficient monitoring programs in contaminated areas, the integration of data obtained with different techniques is useful, we present a preliminary work aimed at investigating the possibility of using biofilm as a potential integrative bioindicator to assess the contamination levels and distribution in the surface water of the contaminated area around the chemical plant located along the Toce River. 

## 2. Materials and Methods

### 2.1. Study Area

**Figure 1:**
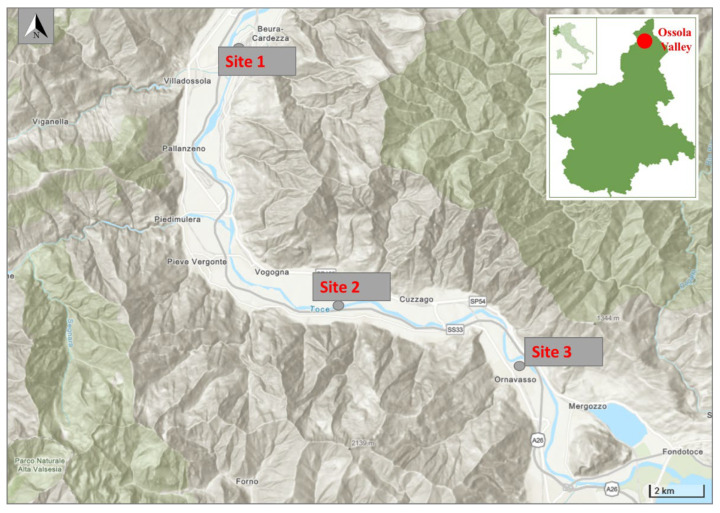
Study area and sites of the biofilm sampled in the Toce River (Ossola Valley).

This research was carried out in Ossola Valley located in Piedmont, Italian central Alps, which borders the Pennine and Lepontine Alps, Switzerland, and Lake Maggiore. The Toce River, an Alpine river, stretches the length of the Ossola Valley flowing into Lake Maggiore for 84 km, and the catchment area is about 1784 km^2^ [18]. We collected biofilm at three selected sampling points: a point located north of the chemical plant, one located south of the plant, and a third point located at the mouth of the Toce River, where it flows into Lake Maggiore. The studied sites coordinates are reported in Table 1 and shown in Figure 1.

### 2.2. Biofilm Collection and Samples Preparation

Biofilm samples were collected following the European standard protocol [19] for the benthic diatoms from rivers. At each site, a 50 m long river stretch was investigated, and biofilm was collected at 4 points from the natural hard surface, i.e., cobbles and stones, completely submerged at a depth of 20–30 cm from 4 locations around the river. Cobbles and stones were moved from the riverbed, and the surface was scraped from each stone, placed into a falcon filled with river water, and kept in a cool dry place. In the laboratory, the samples were centrifuged at 3500 rpm, and aliquots of the pellets were frozen, freeze-dried, or dried at 50 °C for two days as indicated below.

### 2.3. Diatoms Identification

The samples for diatom identification were treated with hot hydrogen peroxide and hydrochloric acid following standard procedures [20] and finally mounted using Naphrax (Brunel Microscopes Ltd., Chippenham, UK) on permanent slides for species identification (Zeiss Axiolab, magnification 1000×). Taxonomic identification was based on Krammer and Lange-Bertalot [21], Lange-Bertalot [22], Krammer [23,24,25], Lange-Bertalot et al. [26], and Cantonati et al. [27] integrated with the paper on the Achnanthidium minutissimum species complex by Potapova and Hamilton [28]. For each sample, a minimum of 400 valves were identified using a Zeiss Axiolab microscope (Gottingen, Germania), and results were expressed as relative abundances (%). Subsequently, the α-diversity and heterogeneity of the diatomic communities for each sampling site were evaluated on the basis of the Shannon index [29] and Evenness [30], respectively.

The ecological status was assessed via calculation of the Intercalibration Common Metrics Index ICMi [31], which is calculated as the mean of the ecological quality ratio (EQR) of two existing indices, IPS [32] and TI [33].

### 2.4. Community Taxonomic Composition

Sub-samples from each site were also preserved with Lugol’s Iodine [19]; the reagents were all of analytical grade by VWR (Milan, Italy). Taxonomic composition of the non-diatom community was analyzed under a Leica inverted microscope (Leica Srl, Milan, Italy). Sedimentation chambers were filled with diluted sediment samples and evaluated at 400×–1000× magnification following the Üthermol method [34] and Lund [35]. Further, an aliquot from each sample was used for the identification of algae (except for diatoms), in temporary slides at 1000× magnification under lightfield, darkfield, and phase contrast microscopy techniques to characterize them morphologically. Image analyses were also used to perform identification to the lowest taxonomical level (species or genus). 

### 2.5. Quantification of Carotenoids and Chlorophylls in Biofilm

The carotenoids were extracted from the freeze biofilm using acetone 90%. Spectrophotometric evaluation of chlorophylls a and pheophytin was performed following the method reported by Steinman and Lamberti [36]. The reagents were all of analytical grade by VWR (Milan, Italy). The data from each experiment represent the mean (± standard deviation, SD) of three replicates.

HPLC analysis, on the same extracts, was performed with a Thermo Fisher Scientific (Waltham, MA, USA) “UltiMate LC System” composed of a UV/VIS detector, and a Photodiode Array BioLC detector [37]; reagents were HPLC Lichrosolv by VWR (Milan, Italy). The detailed method is in Supplementary materials Method M1.

### 2.6. Quantification of Metals and Macroions in Biofilm

For the determination of elements of interest (As, Al, Ba, Ca, Cr, Cu, Fe, K, Mg, Mn, Ni, P, Pb, Zn), aliquots of 150 mg of dried biofilm samples were digested by a microwave digestor system (Anton Paar MULTIWAVE-ECO) in Teflon tubes filled with 10 mL of 65% of nitric acid by employing a one-step temperature ramp (the temperature is increased to 180 °C in 20 min and maintained for 10 min). After 20′ of cooling time, the mineralized samples were transferred to polypropylene test tubes, and the final volume was registered. Then samples were properly diluted (1:100) with 1.3 M of nitric acid in MILLI-Q water, and the concentration of elements was measured via inductively coupled plasma mass spectrometry (BRUKER Aurora-M90 ICP-MS). To check the nebulization performance, an aliquot of 2 mgL^−1^ of an internal standard solution (72Ge, 89Y, 159Tb) was added to the samples and to the calibration curve to give a final concentration of 20 µgL^−1^.

Typical polyatomic analysis interferences were removed using CRI (Collision-Reaction-Interface) with an H_2_ flow of 70 mLmin^−1^ flown through a skimmer cone [38]. All reagents were of analytical grade by VWR (Milan, Italy). The data from each experiment represent the mean (± standard deviation, SD) of three replicates.

### 2.7. Determination of Hg in Biofilm

Freeze-dried biofilm was homogenized using a Retsch MM2000 ball mill (Retsch Technology GmbH, Haan, Germany). Total Hg concentration in the biofilm was determined via thermal decomposition, amalgamation, and atomic absorption spectrometry according to the US-EPA method 7473 [39], using an automated mercury analyzer (AMA-254, FKV Srl, Bergamo, Italy). The instrument detection limit is 0.01 ng Hg, while the working range is 0.05 to 600 ng Hg. The limit of quantification (LOQ), calculated as ten times the standard deviation of the blank and considering a sample mass (plant tissue) of 25 mg, is 0.009 mg kg^−1^.

For accuracy evaluation, the certified reference material BCR-CRM061 aquatic moss powder (Community Bureau of Reference, Institute for Reference Materials and Measurements, Geel, Belgium) was analyzed (reference value = 0.23 ± 0.02 mg kg^−1^), obtaining a mean recovery of 103.8 ± 1.1% (n = 3) of certified values. Precision was checked via triplicate analysis and percent coefficient of variation, calculated as the ratio of the standard deviation to the mean of the three analyses of each sample *100, was ≤ 7%, except for the sample collected at Site 1 in October (36%), which was difficult to homogenize.

## 3. Results

### 3.1. Biofilm Characterization

#### 3.1.1. Diatoms Identification

A total of 89 species, belonging to 36 genera, were identified in the three sites in March and October, to the highest taxonomic resolution possible; only 5 of them had a relative abundance greater than 10% (dominant species) and 9 greater than 5% but less than or equal to 10% (sub-dominant species). The most abundant genera were Nitzschia (15), Achnanthidium and Navicula (8 each), Fragilaria and Gomphonema (7 each), Encyonema and Cocconeis (5 each), while all other genera were represented by less than 5 taxa. About 19% of the taxa identified are included in the Red List [40]: in most cases, these are species whose abundance and frequency are estimated to be decreasing, but species defined as “in danger of extinction” were also present (approximately 3%) (Table S1 (Supplementary material)). 

The most common diatoms were achnanthoid taxa (mainly Achnanthidium minutissimum and Achnanthidium subatomus), Diatoma mesodon, Encyonema minutum, Navicula cryptotenella, and nitzschioid taxa (mainly Nitzschia dissipata, Nitzschia fonticola and Nitzschia heufleriana). A. minutissimum and A. subatomus were also the most abundant taxa, as they reached a relative abundance of > 30 % in several samples. Both samples collected at Site 3 and sample collected in autumn from Site 1 in were also rich in nitzschioid taxa (relative abundance > 20%). Autumn samples also showed higher diversity than spring samples, and some frustules (1.5%) appeared with the teratogenic morphotype. Evenness was similar in Site 2 and Site 3 but higher at Site 1. The assessment of the quality status based on the application of the Intercalibration Common Metrics Index (ICMi) is reported in Table 2. The index attributed a high ecological class to all samples taken in spring and to the one collected in autumn at Site 2, a moderate quality at Site 1, and a poor quality to the autumn sample from Site 3.

#### 3.1.2. Biofilm Taxonomic Identification of Non-Diatom Algal Community

Algae taxa composition of non-diatom community was obtained by microscope evaluation of samples collected at the three sites studied in March and October. A total of 46 taxa of the non-diatom algal community were identified, mainly formed by Cyanobacteria and Chlorophyta at the sampled stations. Cyanobacteria were well represented by chrooccocales and filamentous genera. The most abundant group found was the Cyanobacteria, with 22 taxa and 14 genera, followed by Chlorophyta, with 20 taxa and 17 genera (Table S2). The most representative species among Cyanobacteria was *Aphanothece minutissima*, found in all sampling stations both in March and in October, together with *Chamaesiphon* and *Leptolyngbya* genera. The chroococcalean species *Bacularia* cfr. *gracilis* was found only at Site 2 in October. Chlorophyta sensu lato was dominant, with 10 orders mostly represented by 6 genera of Sphaeropleales and 3 genera of Desmidiales and Ulotrichales.

**Figure 2:**
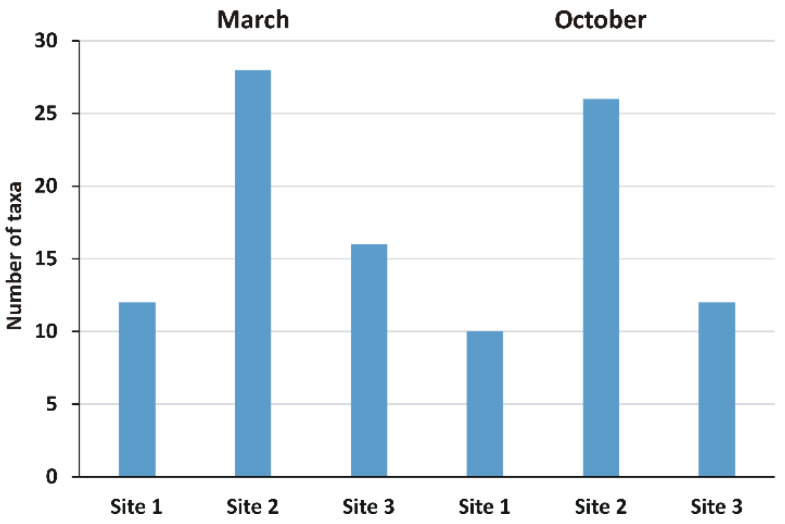
Number of non-diatom algal taxa observed in biofilm in March and in October. Site 1 (Villadossola), Site 2 (Bosco Tenso), Site 3 (Ornavasso).

The green algae *Pediastrum boryanum* and desmidiales *Closterium dianae* and *Tetraselmis cordiformis* were found only at Site 2 in October 2019. Chryptophyceae, Xanthophyceae, Euglenophyceae, and Chrysophyta were represented by one taxon each. Site 2 was the richest with 28 species in March and 26 in October, followed by Site 3 with 16 species in March, Site 1, and Site 3 both with 12, respectively, in March and October, while Site 1 resulted in the poorest in species with 10 in October. The Amoebozoa represented by *Galeripora discoides* and *Euglypha crenulate* species were found at site 1 in October. Figure 2 reports the total number of taxa observed in each sample site in March and October; the full list of the identified taxa is reported in Table S2. 

### 3.2. Quantification of Carotenoids and Chlorophyll in the Biofilm

**Figure 3:**
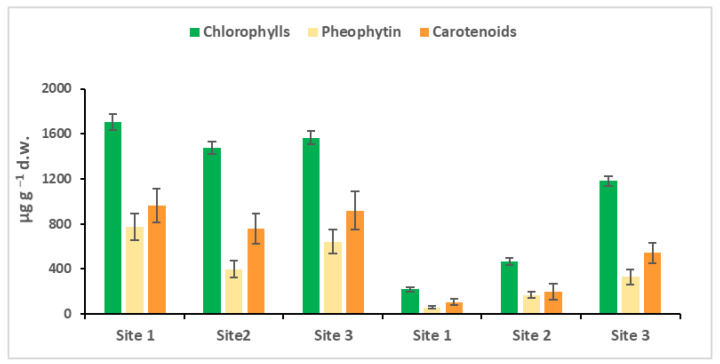
Chlorophyll, pheophytin, and carotenoid quantification in each biofilm sample in March and October. Site 1 (Villadossola), Site 2 (Bosco Tenso), Site 3 (Ornavasso). Dry weight = d.w.

The spectrophotometric quantifications of the chlorophylls, their degradation products, the pheophytin, and carotenoids of the biofilm are shown in Figure 3. In March, the environmental conditions, temperature, light and nutrients in the Toce River develop the optimal conditions for the diatoms (fucoxanthin), pioneer organisms, as evidenced in Figure 3. The total content of pigments (chlorophyll and carotenoid) in the biofilm at the three sites was very similar, and the only difference was that site 2 showed less pheophytin, the first product of chlorophyll degradation, which means lesser cellular degradation, or a higher cellular turnover. In October, the production of the pigments was much lower, lesser than in March; the behavior was typically in a pre-alpine area, where the water temperature and especially the active photosynthetic irradiance decreased. 

**Figure 4:**
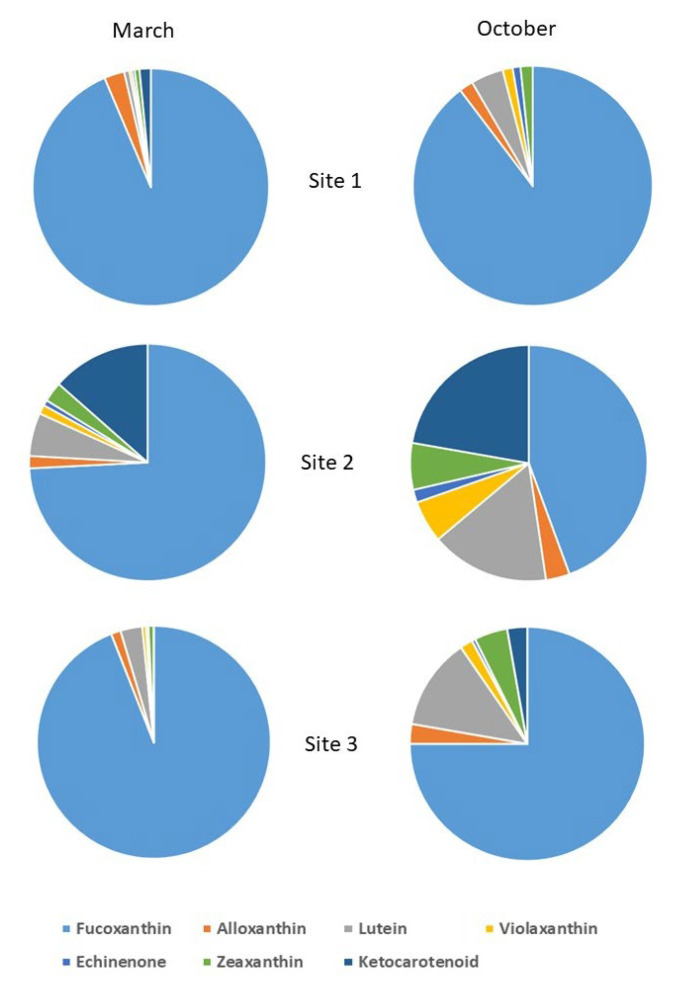
HPLC characterization of carotenoids in each biofilm sample. Carotenoids are reported in the legend with different colors. Site 1 (Villadossola), Site 2 (Bosco Tenso), Site 3 (Ornavasso).

HPLC characterization allowed carotenoid molecule identification in the biofilms (Figure 4). The carotenoids are useful bioindicators of algal taxa [37,41]. Fucoxanthin dominate all samples in March and October in all sites, as confirmed by the microscope identification; diatoms were the principal phytobentonic organisms in the biofilm, which represents a good ecological quality in the mountain streams in March, Table 2. In March, the Site 2 (Bosco Tenso) biofilm was rich in ketocarotenoids, zeaxanthin and echinenone (carotenoids of cyanobacteria), lutein and violaxanthin (carotenoids of chlorophytes), and alloxanthin (carotenoid of cryptophytes), confirmed also by non-diatom taxa identification (Figure 2). In October, at Site 2, lutein (chlorophytes) and ketocarotenoids (cyanobacteria) were in competition with diatoms. In October, at site 3, an increase in lutein (chlorophytes) and ketocarotenoids and zeaxanthin (cyanobacteria) was observed. 

### 3.3. Quantification of Metals and Macroions in the Biofilm

#### 3.3.1. ICP-MS Quantification in Biofilm Samples

**Figure 5:**
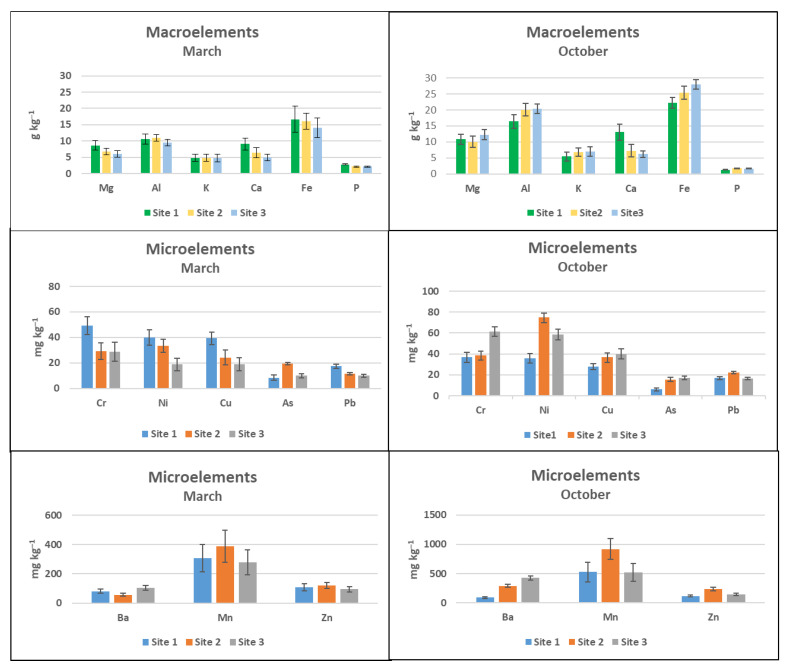
ICP-MS macroelements and microelements in the biofilm. Site 1 (Villadossola), Site 2 (Bosco Tenso), Site 3 (Ornavasso). Data reported on dry weight.

Macroelements measured in the biofilms were in the range of g kg^−1^ in March and in October at the three sites (Figure 5). In October, the amount of aluminum and iron was higher than in March, but with the same trend, it means that the water transported more elements. The behavior of the microelements was different. In March, chromium, nickel, and copper were higher at Site 1 than at Site 2 and Site 3. In October, the behavior changed and the total amount of microelements increased, but the amount of nickel increased at Site 2 and that of chromium at Site 3. Arsenic and lead were present but almost constant in March and in October. Barium, manganese, and zinc showed the same behavior in March and in October in the three sites. 

#### 3.3.2. Hg in the Biofilm

**Figure 6:**
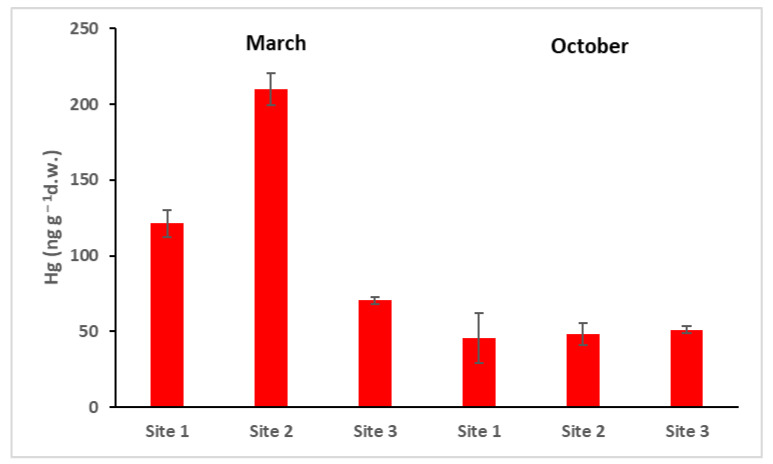
Total Hg in the biofilm. Site 1 (Villadossola), Site 2 (Bosco Tenso), Site 3 (Ornavasso). Dry weight = d.w.

Total Hg measured in the biofilm ranged from 50 ngg^−1^ to 200 ngg^−1^ dry weight (Figure 6). Site 1 was located upstream of the industrial site of the chlor-alkali plant, but as evidenced in a previous paper on the dispersion of Hg in Ossola Valley [17], Hg can be transported by the wind also to the north, and from the Toce River to the south. The complex equilibrium of Hg in the different environmental compartments (air, water, and soil) leads to a distribution and deposition everywhere, even far from the primary sources of pollution. 

## 4. Discussion

Data reported in this preliminary work are intended to present a case study, to support the evidence and the importance of investigating the role of biofilm as an additional bioindicator. Even if diatoms are the dominant species in the phytobentonic component, the three sites show significant differences that are particularly evident in the month of October. Site 2, Bosco Tenso, in March and October presents a peculiar behavior, high ecological quality of diatoms, and higher biodiversity within the algal community as shown by a more diverse carotenoid composition, compared to Site 1 and Site 3. At the same time, biofilm showed higher Hg content in March, while a higher concentration of nickel, chromium, aluminum, and iron was shown in October, being located downstream of the polluted industrial area. Relatively higher Hg concentration might be responsible for the accumulation of ketocarotenoid at this site. Diatoms [5,42] and cyanobacteria [43,44] can produce, with metals, or higher ions molecules EPS, a useful protection system that allows bacteria to adapt more easily to environmental changes. This external matrix protects organisms by binding water, and with electrostatic interactions, binding metals, macroions, and nutrients, which are used by different organisms when needed or left outside the cells without damage from oxidative stress [1]. 

This study also evidenced the relevance of studying the biofilm not only for the identification of the taxa present but also as a bioindicator of accumulation of metals and macroions in water. In the case of the Toce River, significant concentrations of some microele-ments may be expected because of the presence in the watershed of mafic–ultramafic rocks rich in nickel and chromium [45] and arsenopyrite rich in arsenic [8]. However, biofilm analysis can be used as an effects approach to assess the bioavailability of these metals for the aquatic biota. Biofilm could also be useful in studying the accumulation of PFASs and other emerging contaminants or microplastics, as reported by the recent literature [6,46]. Pollutants dispersed in water, soil, and air aerosol that fall back into the water and are transported by the stream can be trapped into the polymeric matrix of the biofilm: measuring them can help us to improve our knowledge of their environmental dispersion.

## 5. Conclusions

Results reported in this preliminary case study on the Toce River evidence the strong seasonal pattern of metals’ and macroions’ distribution in water and highlight the importance of investigating the role of biofilm as an additional bioindicator to enhance the water quality monitoring program in the river. A more detailed monitoring plan, based on monthly analysis, would clarify the distribution pattern of pollutants in the area and improve our knowledge of the adaptive responses of primary producers in the biofilm.

## Figures and Tables

**Table 1:** Site coordinates.

Site	Location	Longitude	Latitude
1	Villadossola	08°16′59.04” E	46°04′35.31” N
2	Bosco Tenso	08°19′57.47” E	45°59′38.59” N
3	Ornavasso	08°25′05.18” E	45°58′35.32” N

**Table 2:** Assessment of the ecological status of the Toce River, based on biofilm diatom community.

		March			October		
Index	Ref. *	Site 1	Site 2	Site 3	Site 1	Site 2	Site 3
IPS	19.6	17.2	17.8	16.6	12.8	17.4	11.4
TI	1.2	1.8	1.5	1.8	2.48	1.8	2.83
RQE_IPS		0.8776	0.9082	0.8469	0.6531	0.8878	0.5816
RQE_TI		0.7857	0.8929	0.7857	0.5429	0.7857	0.4179
ICMi		0.8316	0.9005	0.8163	0.5980	0.8367	0.4997
Ecological Quality		high	High	high	moderate	high	poor

* Ref: reference value attributed to the rivers belonging to the “Alpine siliceous macrotype”.

## Data Availability

Not applicable.

## Supplementary Materials

The following supporting information can be downloaded at: https://www.mdpi.com/article/10.3390/toxics10120791/s1, Method M1: Quantification of carotenoids and chlorophylls in biofilm; Table S1: List of diatomic species; Table S2: List of non-diatom taxa.
