# Supplementary material for: Biofilm and Rivers: The Natural Association to Reduce Metals in Waters
Source: Toxics. 2022 Dec 15;10(12):791. doi: 10.3390/toxics10120791 (PMC9786160; doi:10.3390/toxics10120791)
Supplement: Supplementary file 1 [file toxics-10-00791-s001.zip › toxics-2102569-supplementary.pdf]

Supplementary materials

Biofilm and rivers: the natural association to reduce metals in waters.

Method M1. Quantification of carotenoids and chlorophylls in biofilm

Algal and bacterial pigments are extracted (overnight, at 4 °C, in the dark and under nitrogen) from ca. 1-2 g wet biofilm using 90% acetone and then centrifuged at 4000 rpm for 10 min in 15 ml glass centrifuge tubes. The supernatant is used to measure total pigment concentration (UV-VIS spectrophotometer, SAFAS UVmc2) and specific carotenoids (HPLC Ultimate 3000, Thermo Fisher Scientific).

SAFS UV-Vis mc2 is a dual-band spectrophotometer, the spectra are recorded in the range 350-800nm and the Chlorophyll Derivatives (CD) and Total Carotenoids (TC) are calculated as reported in Lami et.al [32]. Specific carotenoids and chlorophylls are determined by ion pairing and reverse-phase HPLC. Pigment concentrations are expressed as nanomoles per gram of organic matter. The ion pairing (PIC A) allows for greater resolution of the dephytolated acidic chloropigments (Chl c, chlorophyllide a, and pheophorbide a).

The HPLC system consists of an autosampler (Ultimate 3000), a quaternary pump (P680), a thermostated column oven (TCC100), and a DAD detector (Ultimate 3000). The chromatogram is acquired at 460 nm and 656 nm for carotenoids and chloropigments, respectively. The whole spectra, for peak identification, are also recorded. The column is a C18 ODS column (5 µm particle size; 250 mm x 4.6 mm i.d.). After sample injection (100 µl of acetone extract, a gradient program that ramped from 85% mobile-phase A (80:20, by vol. methanol: aqueous solution of 0.001 M PIC A ion-pairing and 0.001 M propionic acid) to 100% mobile-phase B (60:40, acetone: methanol) in 30 min with a hold for 20 min provided sufficient resolution of all pigments of interest. Flow rates from 1 ml min-1 to 2 ml min-1. The column is re-equilibrated between samples by linear ramping to 85% mobile-phase A for 5 min and maintenance for 10 min before sample injection. With this procedure, we are able to separate zeaxanthin from lutein and ß-carotene from pheophytin a. Analysis of replicates samples yielded a C.V. of 4.5%- 11.5%, depending on pigments.

Table S1. List of diatomic species. Species expressed as presence/absence identified in the 6 samples analyzed and risk status according to the Red List (Hofmann et al., 2018). 0-not classified, 3-Highly endangered, 4-Threatened of extinction, 5-Risk not estimated, 7-Early alert, 8-Not endangered, 9-Not enough data.

		March			October		
**Species**	**Red List**	**Site 1**	**Site 2**	**Site 3**	**Site 1**	**Site 2**	**Site 3**
Achnanthidium eutrophilum (Lange-Bertalot) Lange-Bertalot	8	x	x				
Achnanthidium lineare W.Smith	3		x	x	x	x	
Achnanthidium minutissimum (Kützing) Czarnecki	0	x	x	x	x	x	x
Achnanthidium pyrenaicum (Hustedt) Kobayasi	8	x	x		x	x	
Achnanthidium straubianum (Lange-Bertalot) Lange-Bertalot	8				x		
*Achnanthidium s*ubatomus (Hustedt) Lange-Bertalot	7	x			x	x	x
Adlafia minuscula var. *minuscula* (Grunow) Lange-Bertalot	8					x	x
Adlafia muralis (Grunow) Li & Qi	8		x				
Amphora aequalis Krammer	4			x	x		x
Amphora inariensis Krammer	8		x				
Amphora pediculus (Kützing) Grunow	8	x		x	x	x	x
Caloneis silicula (Ehrenberg) Cleve	8						x
Cocconeis euglypta Ehrenberg	8				x	x	x
Cocconeis placentula Ehrenberg	9	x	x		x	x	
Cocconeis pseudolineata (Geitler) Lange-Bertalot	8	x	x		x	x	
Craticula buderi (Hustedt) Lange-Bertalot	8						x
Cyclotella distinguenda Hustedt	8					x	
Cymatopleura solea (Brébisson) W. Smith	8						x
Cymbella C.Agardh	0	x				x	
Cymbella excisa Kützing	8	x			x	x	x
Cymbopleura subaequalis var. *subaequalis* (Grunow) Krammer	4				x		
Denticula tenuis var. *tenuis* Kützing	0				x		
Diatoma ehrenbergii Kützing	8	x	x		x	x	x
Diatoma mesodon (Ehrenberg) Kützing	8	x	x	x	x		
Diatoma moniliformis Kützing	8	x				x	
Encyonema minutum (Hilse in Rabh.) D.G. Mann	8	x	x	x	x	x	x
Encyonema reichardtii (Krammer) D.G. Mann	8	x					
Encyonema silesiacum (Bleisch) D.G. Mann	8	x	x	x		x	x
Encyonema ventricosum (Kützing) Grunow	8				x		
Epithemia F.T. Kützing	0					x	
Eunotia C.G. Ehrenberg	0					x	
Eucocconeis laevis (Østrup) Lange-Bertalot	7		x		x		
Fragilaria gracilis Østrup	8		x	x		x	
Fragilaria pararumpens Lange-Bertalot, G.Hofmann & Werum	8						x
Fragilaria pectinalis (O.F.Müller) Lyngbye	9		x	x			x
Fragilaria perdelicatissima (W.Smith) Lange-Bertalot & Van de Vijver	7			x			
Fragilaria perminuta (Grunow) Lange-Bertalot	8	x		x			
Fragilaria rumpens (Kützing) G.W.F.Carlson	8			x			
Fragilaria vaucheriae (Kützing) Petersen	8	x	x	x		x	
Gomphonema C.G. Ehrenberg	0	x	x			x	x
Gomphonema angustivalva E. Reichardt	9				x		x
Gomphonema elegantissimum Reichardt & Lange-Bertalot	8				x		

Gomphonema innocens Reichardt	8				x		
Gomphonema pseudotenellum Lange-Bertalot	5		x				
Gomphonema pumilum var. *rigidum* Reichardt & Lange-Bertalot	8	x	x				x
Gomphonema saprophilum (Lange-Bertalot & Reichardt) Abarca, R. Jahn, J. Zimmermann & Enke	8	x	x				
Hannaea arcus (Ehr.) Patrick	7		x	x			
Hippodonta capitata (Ehr.) Lange-BertalotMetzeltin & Witkowski	8						x
Hippodonta pumila Lange-Bertalot, Hofmann & Metzeltin	8					x	
Melosira varians Agardh	8			x		x	x
Navicula J.B.M. Bory de St. Vincent	0					x	
Navicula antonii Lange-Bertalot	8				x	x	x
Navicula caterva Hohn & Hellerman	8				x		
Navicula cryptotenella Lange-Bertalot	8		x	x	x	x	x
Navicula radiosa var. *radiosa* Kützing	8				x	x	x
Navicula reinhardtii (Grunow) Grunow	8	x					
Navicula splendicula Van Landingham	5				x		
Navicula subalpina Reichardt	4		x	x	x	x	x
Nitzschia A.H. Hassall	0		x				x
Nitzschia acidoclinata Lange-Bertalot	7					x	
Nitzschia alpinobacillum Lange-Bertalot	3			x			
Nitzschia archibaldii Lange-Bertalot	8						x
Nitzschia dealpina Lange-Bertalot & Hofmann	4			x	x		
Nitzschia dissipata subsp. *dissipata* (Kützing) Grunow	8	x	x	x	x		x
Nitzschia fonticola Grunow	8	x	x	x		x	x
Nitzschia heufleriana var. *heufleriana* Grunow	8	x	x	x	x		x
Nitzschia inconspicua Grunow	9			x			x
Nitzschia lacuum Lange-Bertalot	5					x	
Nitzschia linearis (Agardh) W.M.Smith	8				x		
Nitzschia palea var. *palea* (Kützing) W.Smith	8		x	x	x		x
Nitzschia palea var. *debilis* (Kützing) Grunow	8			x	x	x	x
Nitzschia palea var. *tenuirostris* Grunow	8				x	x	x
Nitzschia recta Hantzsch ex Rabenhorst	8						x
Pantocsekiella comensis (Grunow) K.T. Kiss & Ács	5					x	
Placoneis pseudanglica (Lange-Bertalot) Cox	8					x	
Planothidium dubium (Grunow) Round & Bukhtiyarova	8		x	x			x
Planothidium frequentissimum (Lange-Bertalot) Round & Bukhtiyarova	8					x	
Planothidium lanceolatum (Brébisson) Lange-Bertalot	8				x		
Psammothidium bioretii (Germain) Bukhtiyarova & Round	8			x	x	x	
Reimeria sinuata (Gregory) Kociolek & Stoermer	8				x	x	x
Rhoicosphenia abbreviata (C.Agardh) Lange-Bertalot	8	x					
Rossithidium pusillum (Grunow) F.E.Round & Bukhtiyarova	3	x					
Sellaphora pupula (Kützing) Mereschkowksy	9						x
Staurosira (C.G. Ehrenberg) D.M. Williams & F.E. Round	0			x			
Staurosira venter (Ehrenberg) Cleve et Moeller	8					x	
Staurosirella D.M. Williams & F.E. Round	0			x	x	x	
Stephanodiscus alpinus Hustedt	8					x	
Tabellaria ventricosa Kützing	4					x	
Ulnaria acus (Kützing) Aboal	8			x			

Table S2. List of non-diatom taxa. Data collected from 3 sample sites in the Toce River in March and October 2019.

	**March**			**October**		
**Species**	**Site 1**	**Site 2**	**Site 3**	**Site 1**	**Site 2**	**Site 3**
**Cyanobacteria**						
Aphanocapsa sp.		X		X		X
Aphanocapsa muscicola (Meneghini) Wille	X					
Aphanothece sp.	X	X			X	
Aphanothece minutissima (West) J.Komárková-Legnerová & G.Cronberg	X	X	X	X	X	X
Aphanothece stagnina (Sprengel) A.Braun		X	X	X	X	X
Bacularia cfr. gracilis (Komárek)					X	
Chamaecalyx swirenkoi (Schirschoff) Komárek & Anagnostidis					X	
Chamaesiphon cfr. polonicus (Rostafinski) Hansgirg	X	X			X	X
Chamaesiphon sp.		X				
Chlorogloea sp.		X			X	
Chroococcus sp.		X		X	X	
Gloeocapsa cfr. sanguinea (C.Agardh) Kützing		X				
Gloeocapsa sp.	X	X	X		X	X
Gloeocapsopsis sp.		X	X	X		
Komvophoron sp.			X			
Leptolyngbya sp.	X	X		X	X	
Leptolyngbya olivacea (Kützing ex Hansgirg) Anagnostidis					X	
Merismopedia minima Beck	X	X			X	
Phormidium sp.	X	X	X		X	
Phormidium autumnale Gomont						X
Pseudanabaena sp.	X	X		X	X	
Pseudanabaena mucicola (Naumann & Huber-Pestalozzi) Schwabe		X			X	
**Chlorophyceae** (sensu lato)						
Characium sp.		X			X	
Chlamydocapsa sp.		X			X	
Chlorella sp.					X	X
Cladophora glomerata (Linnaeus) Kützing			X			
Closterium dianae Ehrenberg ex Ralfs		X				
Coelastrum cambricum W.Archer						X
Cosmarium lobatum Börgesen			X			
Cylindrocystis brebissonii (Ralfs) De Bary					X	
Hyalotheca sp.		X	X	X		
Microspora sp.			X			
Pediastrum boryanum (Trup.) Menegh.					X	
Scenedesmus ecornis (Ehrenberg) Chodat		X				X
Scenedesmus aculeolatus Reinsch		X				
Schizomeris sp.			X		X	
Spirogyra sp.			X	X		X
Staurastrum punctulatum Brébisson					X	X
Tetraselmis cordiformis (H.J.Carter) F.Stein					X	
Ulothrix sp.			X	X		
Ulothrix zonata (F.Weber & Mohr) Kützing	X	X	X		X	X
Ulothrix variabilis Kützing		X		X		

**Chryptophyceae**						
Rhodomonas sp.	X	X				X
**Xanthophyceae**						
Tribonema sp.	X		X			
**Euglenophyceae**						
Phacus caudatus Hübner			X			
**Chrysophyta**						
Hydrurus foetidus (Villars) Trevisan		X		X	X	
**Amoebozoa**						
Tubulinea						
Galeripora discoides (Ehrenberg, 1871) González-Miguéns et al., 2021				X		
Euglypha crenulata Wailes				X		
